# Gallotannin from *Bouea macrophylla* Seed Extract Suppresses Cancer Stem-like Cells and Radiosensitizes Head and Neck Cancer

**DOI:** 10.3390/ijms22179253

**Published:** 2021-08-26

**Authors:** Jiraporn Kantapan, Nathupakorn Dechsupa, Damrongsak Tippanya, Wannapha Nobnop, Imjai Chitapanarux

**Affiliations:** 1 Molecular Imaging and Therapy Research Unit, Department of Radiologic Technology, Faculty of Associated Medical Sciences, Chiang Mai University, Chiang Mai 50200, Thailand; jiraporn.kan@cmu.ac.th (J.K.); nathupakorn.d@cmu.ac.th (N.D.); 2 Department of Radiology, Division of Radiation Oncology, Faculty of Medicine, Chiang Mai University, Chiang Mai 50200, Thailand; damrongsak_tip@yahoo.com (D.T.); wannapha.n@cmu.ac.th (W.N.); 3 Northern Thai Research Group of Radiation Oncology (NTRG-RO), Faculty of Medicine, Chiang Mai University, Chiang Mai 50200, Thailand

**Keywords:** radiotherapy, chemoradiotherapy, radiosensitizer, cancer stem cells, head and neck cancer, apoptosis, Maprang seed extract, gallotannin, pentagalloyl glucose

## Abstract

Cancer stem cells (CSCs) play a critical role in radiation resistance and recurrence. Thus, drugs targeting CSCs can be combined with radiotherapy to improve its antitumor efficacy. Here, we investigated whether a gallotannin extract from *Bouea macrophylla* seed (MPSE) and its main bioactive compound, pentagalloyl glucose (PGG), could suppress the stemness trait and further confer the radiosensitivity of head and neck squamous cell carcinoma (HNSCC) cell lines. In this study, we evaluate the effect of MPSE or PGG to suppress CSC-like phenotypes and radiosensitization of HNSCC cell lines using a series of in vitro experiments, tumorsphere formation assay, colony formation assay, apoptosis assay, and Western blotting analysis. We demonstrate that MPSE or PGG is able to suppress tumorsphere formation and decrease protein expression of cancer stem cell markers. MPSE or PGG also enhanced the radiosensitivity in HNSCC cells. Pretreatment of cells with MPSE or PGG increased IR-induced DNA damage (γ-H2Ax) and enhanced radiation-induced cell death. Notably, we observed that pretreatment with MPSE or PGG attenuated the IR-induced stemness-like properties characterized by tumorsphere formation and the CD44 CSC marker. Our findings describe a novel strategy for increasing therapeutic efficacy for head and neck cancer patients using the natural products MPSE and PGG.

## 1. Introduction

Radiotherapy is a mainstay and an affordable treatment choice for head and neck squamous cell carcinoma (HNSCC). However, radiotherapy is also associated with side effects, particularly the unavoidable development of cancer cell resistance (extrinsic resistance) to radiation exposure [1,2]. Tumor radioresistance, even intrinsic- or extrinsic-resistance, is still the main obstacle to therapeutic success. These radioresistant cells lead to cancer relapse, metastasis, poor prognosis, and poor patient outcome. It is believed that a small subset of cancer stem cells (CSCs) emerge that are capable of self-renewal, differentiation, plasticity, therapy resistance, and high tumorigenicity. CSCs that exhibit such behavior are most likely to be responsible for tumor radioresistance [3]. CSCs have been identified in many types of solid tumor including breast cancer, lung cancer, prostate cancer, and, especially, head and neck squamous cell carcinoma [4,5,6]. Notably, recent studies revealed that anti-cancer therapy (radio/chemotherapy) induced the transformation of non-stem cancer cells to cancer stem cells, enabling them to re-establish the tumors and produce metastasis [7,8]. These findings suggest that anti-cancer therapy not only kills cancer cells but also has the side effect of directly inducing the dynamic transforming non-stem cells into cancer stem cells, possibly contributing to tumor recurrence and metastasis. Therefore, drugs targeting cancer stem cells or preventing reprogramming of non-stem cancer cells can be combined with anti-cancer therapy to improve its antitumor efficacy.

Signal transducer and activator of transcription 3 (STAT3), a transcription factor for survival and proliferation, is essential to survival and maintains tumorigenesis in many cancers [9]. Aberrant activation of STAT3 has been well characterized in HNSCC mainly due to abnormal signaling of various growth factor receptors [10]. The activation of STAT3 contributes to cancer cell proliferation, dedifferentiation, invasion, and angiogenesis [11]. Therefore, targeting STAT3 activation has been proposed as a therapeutic target in cancer treatment. It has been reported that attenuated STAT3 activation results in inhibition of cell proliferation and apoptosis induction in many cancer cells. Furthermore, STAT3 has been validated to affect cancer cell sensitivity to radiation. Recent studies have reported that C225, an anti-epidermal growth factor receptor (EGFR) monoclonal antibody, can, in combination with radiation, suppress STAT3 activation results in radiosensitization in head and neck cancer [12]. In addition, the activation of STAT3 is an essential factor in CSC formation, radioresistance, and metastasis in HNSCC cells [13]. The signaling of cytokine IL-6 leading to STAT3 activation to promote CSC-associated *OCT-4* gene expression contributes to the conversion non-CSCs into CSCs [14]. Due to the pivotal role of STAT3 in HNSCC, these cellular signals are potential molecular targets for effective therapy for HNSCC patients.

Maprang (*Bouea macrophylla* Griffith) seed extract, so-called MPSE, a gallotannin with perfectly mixed constituents, has been shown to exert several pharmacological activities, especially anti-cancer activities [15]. Our previous studies revealed that MPSE contains high amounts of active compounds such as 1,2,3,4-6-pentyl-*O*-galloyl-β-*D*-glucose (PGG), ethyl gallate (EG), and gallic acid (GA). MPSE induces increases in intracellular reactive oxygen species (ROS) and subsequently induces cell cycle arrest and apoptosis in human breast cancer cells [16]. A main active compound found in MPSE, PGG has been extensively studied for its anti-cancer properties. Hyo-Jeong L et al. reported that orally administered PGG can inhibit triple-negative breast cancer (TNBC) growth and metastasis. The mechanism by which PGG exerted anti-tumorigenic effects is probably through the inhibition of STAT3 activation, which further inhibits angiogenesis, proliferation, and induced apoptosis in TNBC [17]. Additionally, PGG inhibits STAT3 phosphorylation and induces caspase-mediated apoptosis in prostate cancer cells in vitro and decreases in vivo tumor xenograft growth. The reduced activation of STAT3 results in the downregulation of downstream target Bcl-XL and Mcl-1 and further induced apoptosis [18]. A notable recent report by our team showed that MPSE pretreatment before irradiation in breast cancer cells inhibits the radiation induced epithelial to mesenchymal transition (EMT) process and confers radiosensitivity, probably by attenuating the phosphatidylinositol 3-kinase (PI3K)/protein kinase B (AKT) and mitogen-activated protein kinase (MAPK) pathways [19].

Herein, we extended the efficacy evaluation of MPSE and PGG radiosensitizing activity in the radioresistant cell lines, HPV-negative HNSCC. Our results provide the evidence of a molecular mechanism for the radiosensitizer effect of MPSE and PGG, demonstrating that MPSE and PGG combination with radiotherapy inhibit the radiation-induced CSCs trait, partially in the path, via attenuating the STAT3 activation and increasing therapeutic efficacy in HNSCC cell lines.

## 2. Results

### 2.1. Characterization of 1,2,3,4-6-Pentyl-O-galloyl-β-D-glucose (PGG) as One of the Main Bioactive Compounds Isolated from Maprang Seed Extract (MPSE)

**Figure 1:**
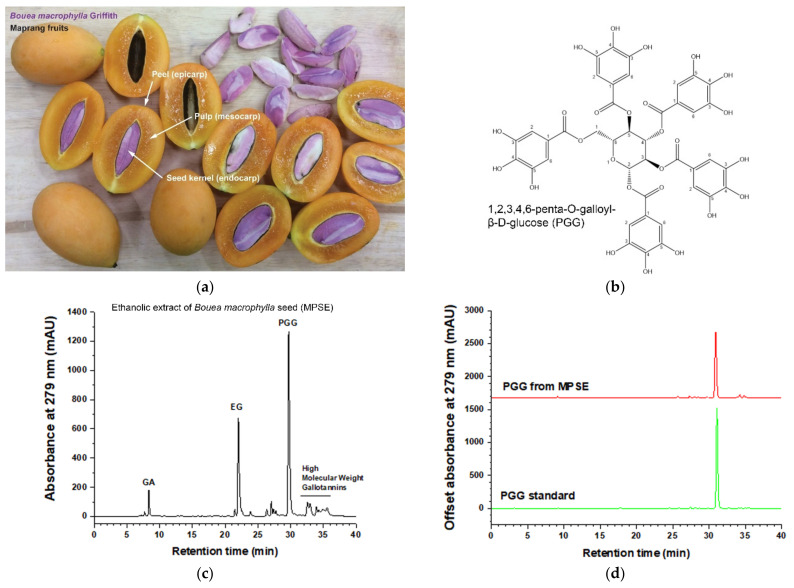
Maprang (*Bouea macrophylla*) fruits and their seed extract phytochemical characterization. (**a**) Maprang (*Bouea macrophylla*) fruits and their three major parts, peel, pulp, and seed kernel. (**b**) Chemical structure of 1,2,3,4-6-pentyl-*O*-galloyl-β-*D*-glucose (PGG). (**c**) HPLC profile of ethanolic extract of Maprang seed extract (MPSE). (**d**) PGG isolated from MPSE (top) and standard PGG samples (bottom).

Before performing the biological test, the chemical profile of Maprang (*Bouea macrophylla* Griffith) (Figure 1a) seed extract, so-called MPSE was determined. MPSE was studied by the high-performance liquid chromatography (HPLC) method to verify the active compound content. We found that MPSE is composed of three major gallotannin compounds, namely 0.5% gallic acid (GA), 36% ethyl gallate (EG), and 50% 1,2,3,4-6-pentyl-*O*-galloyl-β-*D*-glucose (PGG), respectively. While, the other 13.5% composed of unidentified high molecular weight gallotannins were supposedly galloyl glucose species with galloyl moieties greater than five (such as Hexa, Hepta, Octa) [20], and phenolic compounds. (Figure 1c). PGG is a major bioactive compound in MPSE, and its chemical structure is shown in Figure 1b. PGG content in MPSE was analyzed, and it was revealed that PGG had a retention time of 29.6 min, and PGG content was 505.83 µg/mg (Figure 1d).

### 2.2. MPSE or PGG Effectively Inhibits Proliferation and Induces Apoptosis in HNSCC Cell Lines

**Figure 2:**
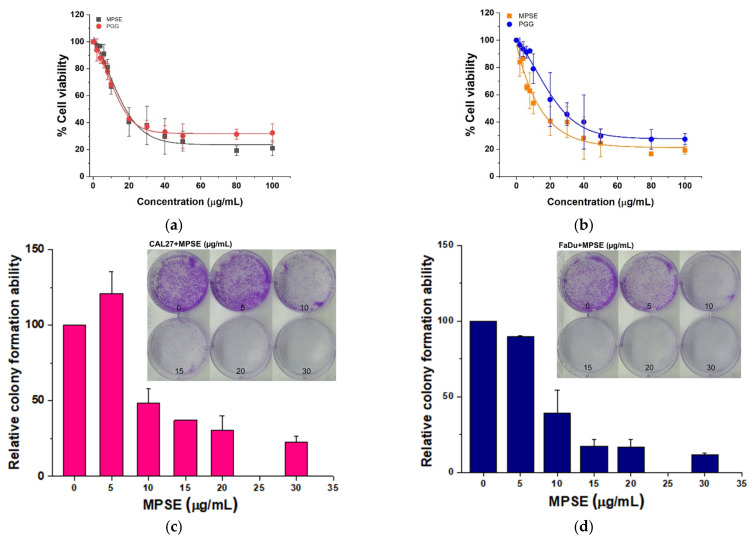

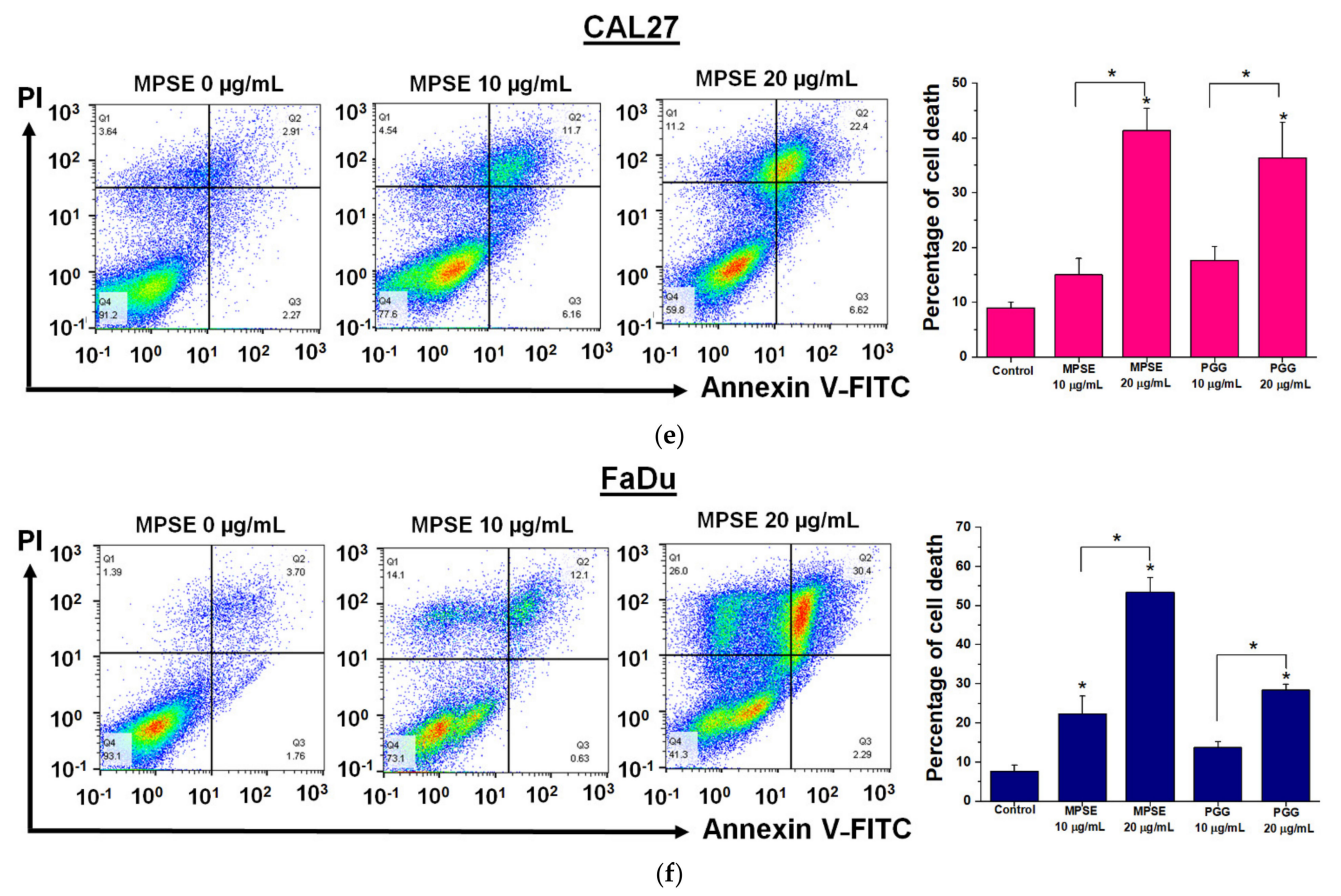
MPSE or PGG suppressed cell proliferation and induced apoptotic cell death in HNSCC cell lines. (**a**) Effect of MPSE or PGG on cell viability of CAL27 cells assessed via MTT assay. (**b**) Effect of MPSE and PGG on cell viability of FaDu cells assessed via MTT assay. (**c**,**d**) The colony-formation ability of HNSCC cell lines treated with different concentrations of MPSE for 48 h, then seeded (1 × 10^4^) in triplicate in a 6-well plate. After two weeks of incubation, formed colonies were counted. The graphs represent the mean ± SD (*n* = 3) of the number of colonies and a representative image of the colony-formation assay (inset panels). (**e**,**f**) Apoptosis induction was analyzed using Annexin V/PI staining after 48 h of treatment with either MPSE or PGG at the indicated concentrations. Left: the representative flow cytometry plots are presented for each cell. Right: quantitated total cell death after treatment with different doses of either MPSE or PGG. All data are presented as mean ± SD (*n* = 3). Statistical significance was evaluated by one-way ANOVA, followed by post hoc Tukey’s multiple comparison test. * *p* < 0.05. MPSE, Maprang seed extract. PGG, pentagalloyl glucose. HNSCC, head and neck squamous cell carcinoma.

We then determined the cytotoxic effect of MPSE or PGG to HPV-negative HNSCC cell lines, CAL27 and FaDu. Cells were incubated with varying concentrations of MPSE or PGG for 48 h, and then cell viability was determined with MTT assay. As shown in Figure 2a,b, MPSE and PGG effectively reduced cell viability of CAL27 and FaDu in a dose-dependent manner. Treatment with MPSE produced a cytotoxic effect with an IC_50_ value for CAL27, and FaDu cells were 18.43 ± 0.80 and 14.52 ± 0.86 µg/mL, respectively. Moreover, PGG showed a cytotoxic effect with an IC_50_ value for CAL27, and FaDu cells were 16.68 ± 1.20 and 26.50 ± 1.46 µg/mL, respectively. It should be noted that MPSE and PGG similarly exhibited a cytotoxic effect against HPV-negative HNSCC cell lines. These results indicate that the standardized gallotannin mixed content in MPSE works synergistically to exhibit anti-proliferation of HNSCC cell lines. Furthermore, we test the effect of MPSE on colony formation. MPSE treatment resulted in inhibition of the colony-forming ability of CAL27 and FaDu cells (Figure 2c,d). Notably, MPSE had a greater effect on FaDu cells compared to CAL27 cells at each respective concentration. In addition, we found that the increase in the population of cells that underwent apoptotic cell death upon treatment with MPSE and PGG corresponded with MPSE- and PGG-induced cytotoxicity in both cell lines (Figure 2e,f). Thus, based on our results, the MPSE and PGG exhibit their anti-proliferative effects in HNSCC cell lines by inducing apoptotic cell death.

### 2.3. MPSE or PGG Effectively Attenuates the Cancer Stem-like Cell Phenotype in HNSCC Cell Lines

**Figure 3:**
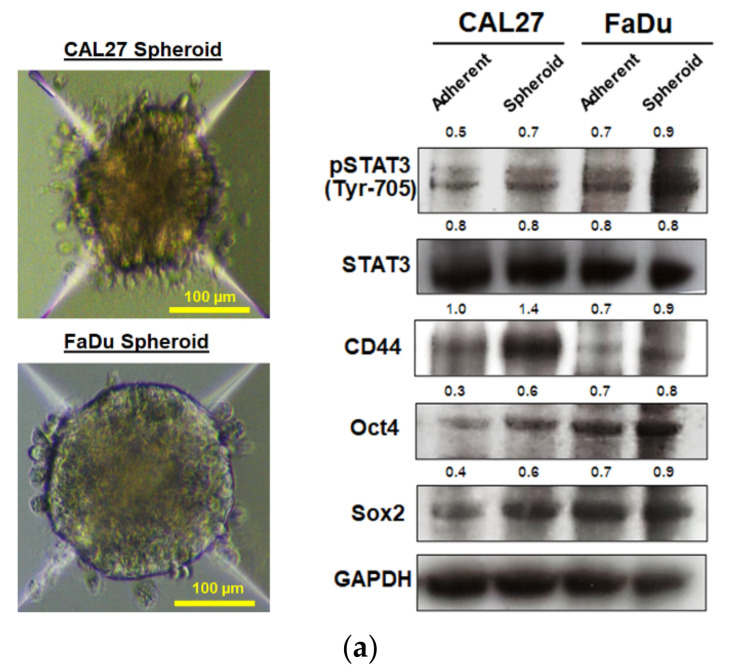

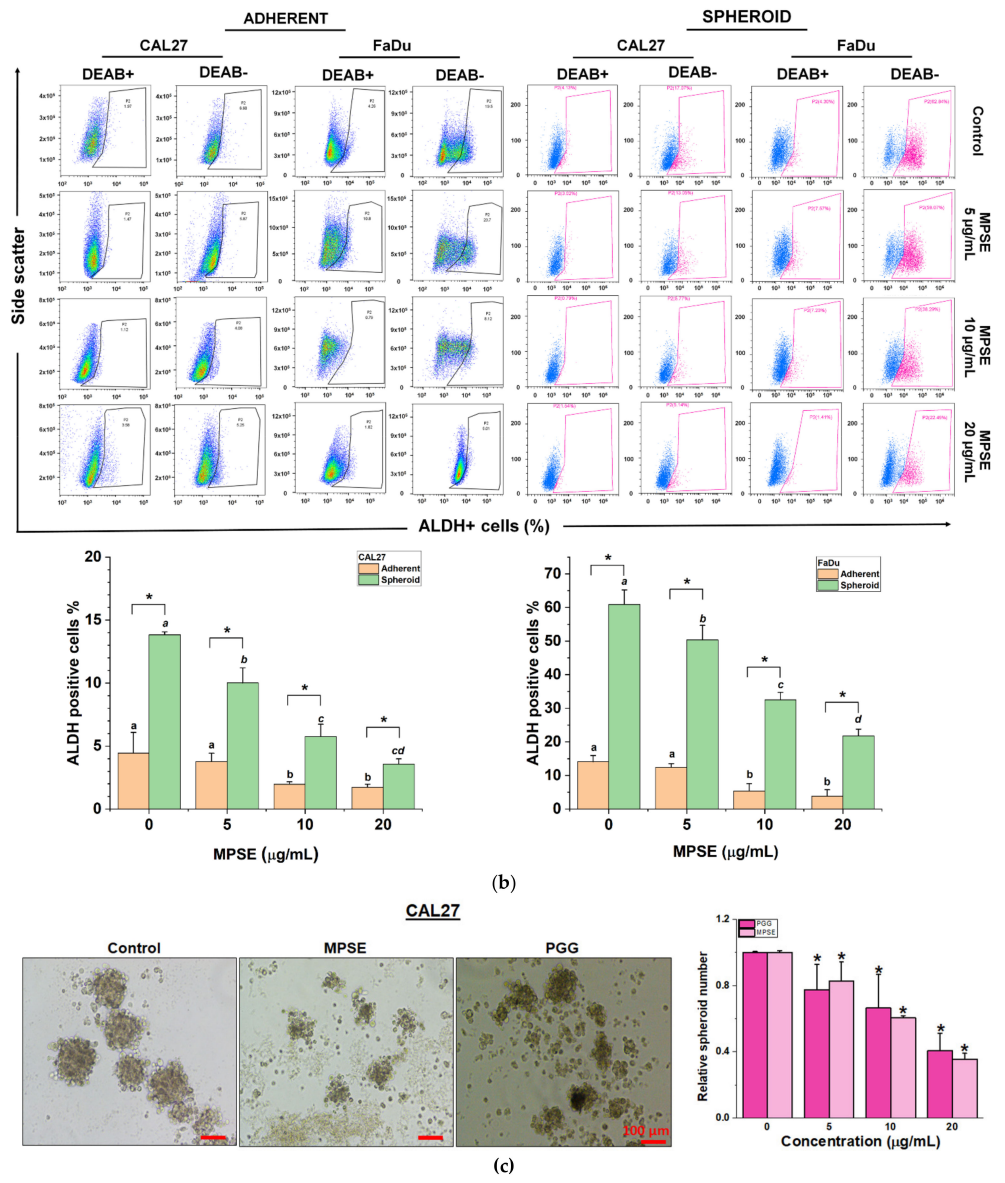

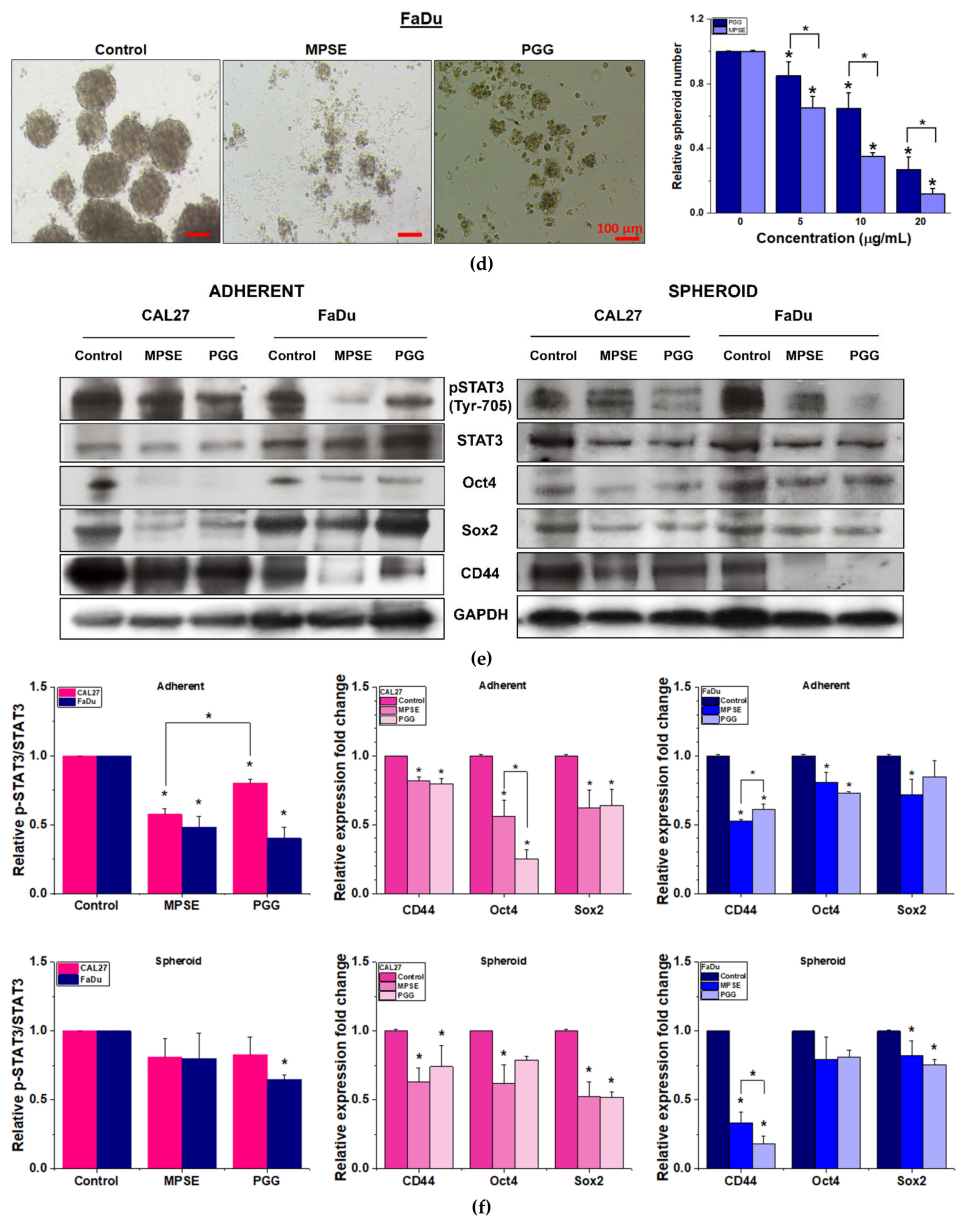
MPSE and PGG suppressed cancer stem-like cell phenotypes in HNSCC. (**a**) Left: representative images of spheroids derived from CAL27 and FaDu cells. Right: the protein expression of stem-cell markers in adherent CAL27 and FaDu cells and their CSC-rich spheroids. The number represents the relative band intensity of p-STAT3, STAT3, Oct4, Sox2, and CD44 was normalized to the band intensity of GAPDH. (**b**) AldeRed ALDH assay by flow cytometry showed decreased ALDH+ population after MPSE treatment. Data was expressed as percent of ALDH+ cells and shown as mean ± SD (*n* = 3). Different letters (abc—adherent; *abc*—spheroid) at the top of columns indicate significant differences at *p* < 0.05 using one-way ANOVA and Tukey’s multiple comparison test. Student’s *t*-test was used to compare the differences between the adherent and spheroid groups, * *p* < 0.05. (**c**,**d**) Tumor sphere formation capacity in CAL27 and FaDu cells. Left: representative images of spheroids treated with either MPSE or PGG. Right: quantification of spheroid numbers derived from CAL27 and FaDu cells after treatment. (**e**) Protein expression of p-STAT3, STAT3, and stem cell markers including, Oct4, Sox2, and CD44 in adherent CAL27 and FaDu cells and the spheroid-derived CSC-rich cells after being treated with 20 µg/mL of either MPSE or PGG, were determined using Western blotting. (**f**) The immunoblot signal intensities were quantified by densitometry. Relative band intensity was normalized to the band intensity of GAPDH. All data show the mean ± SD (*n* = 3). Statistical significance was evaluated by one-way ANOVA, followed by post hoc Tukey’s multiple comparison test. * *p* < 0.05. MPSE, Maprang seed extract. PGG, pentagalloyl glucose. HNSCC, head and neck squamous cell carcinoma.

It is well established that cancer stem cells are responsible for therapeutic resistance and relapse due to their fundamental properties, which are self-renewal and differentiation properties. To investigate whether either MPSE or PGG might suppress the cancer stem-like population in HNSCC, we first generated spheroids from CAL27 and FaDu cells by cultivating them in ultra-low attachment plate under serum-free condition medium. Figure 3a shows that two cancer cell lines exhibit a certain degree of spheroid growth. The CAL27 cells formed small spheres with loose clusters, whereas the FaDu cells showed the best cluster shape, cell–cell adherence, and rounded clusters. As expected, we observed an upregulation of cancer stem cell markers Oct4, Sox2, and CD44 and the phosphorylation of STAT3 in the spheroids. Hence, following confirmation for the enrichment of cancer stem-like cells in spheroids, we used these as a model system to further investigate the effects of either MPSE or PGG on HNSCC stem-like cells. Flow cytometry was performed to examine whether MPSE could decrease ALDH activity in these cells. Intracellular ALDH activity has been identified as a putative marker of cancer stem cells (CSCs) in head and neck cancer cells [21]. The experimental results showed (Figure 3b) that ALDH+ cells ratio was 4.45% and 13.84% in adherent cells and sphere cells in CAL27, and 14.11% and 60.89% in FaDu, respectively. Our results indicated that there was indeed a small amount of ALDH+ cells in both HNSCC cancer cells. In contrast, the proportion of ALDH+ cells in two cell lines showed a significant increase in tumor spheroid culture, indicating that the serum-free suspension culture method can significantly enrich CSCs. In addition, the increasing trend, and the proportion of ALDH+ cells of the FaDu cell line were higher than that of CAL27 cells. Moreover, our results showed that MPSE treatment significantly decreases the population of cells with high ALDH activity, representing the population of CSCs in head and neck cancer cells, in a dose-dependent manner (Figure 3b). To further investigate the effects of either MPSE or PGG in inhibiting the proliferation of the stem cell-like subpopulations in HNSCC cancer, we measured the sphere formation of CAL27 and FaDu cells after treatment. As shown in Figure 3c,d, both the CAL27 and FaDu control cells were able to form aggregates and large-size spheroids. Treatment of the cells with MPSE or PGG significantly interfered with the tumor spheroid formation, and we observed a reduced number of tumor spheroids in a dose-dependent manner.

A previous study suggests that STAT3 signaling pathways are involved in the maintenance of CSC properties in head and neck cancers [13,22]. To elucidate whether either MPSE or PGG effects on self-renewal ability were due to attenuated STAT3 activation, the cells were treated with either MPSE or PGG for 48 h, and the expression levels of phosphorylated STAT3 and CSCs related proteins were determined by Western blot analysis. The results revealed that MPSE and PGG induced a significant decrease in phosphorylated STAT3 protein expression compared with untreated control in both cell lines, while the levels of total STAT3 were not changed. Similarly, stem cell transcription factor Oct4, Sox2, and CD44, a downstream target protein, were significantly reduced following the decline in phosphorylated STAT3 expression in both CAL27 and FaDu cells (Figure 3e,f). A similar trend of results was found for the tumor spheroids derived from both CAL27 and FaDu cells. MPSE and PGG significantly decreased the expression level of stem cell markers CD44 and Sox2, while the phosphorylation of STAT3 and Oct4 is slightly decreased but was not statistically significant in both cell lines. These results suggested that MPSE and PGG could suppress stemness maintenance of head and neck cancer and inhibit cancer stem cell formation with corresponding suppression of cancer stem cell markers.

### 2.4. MPSE or PGG Enhances Radiosensitivity and Induces G2/M Phase Cell Cycle Arrest in HNSCC Cell Lines

**Figure 4:**
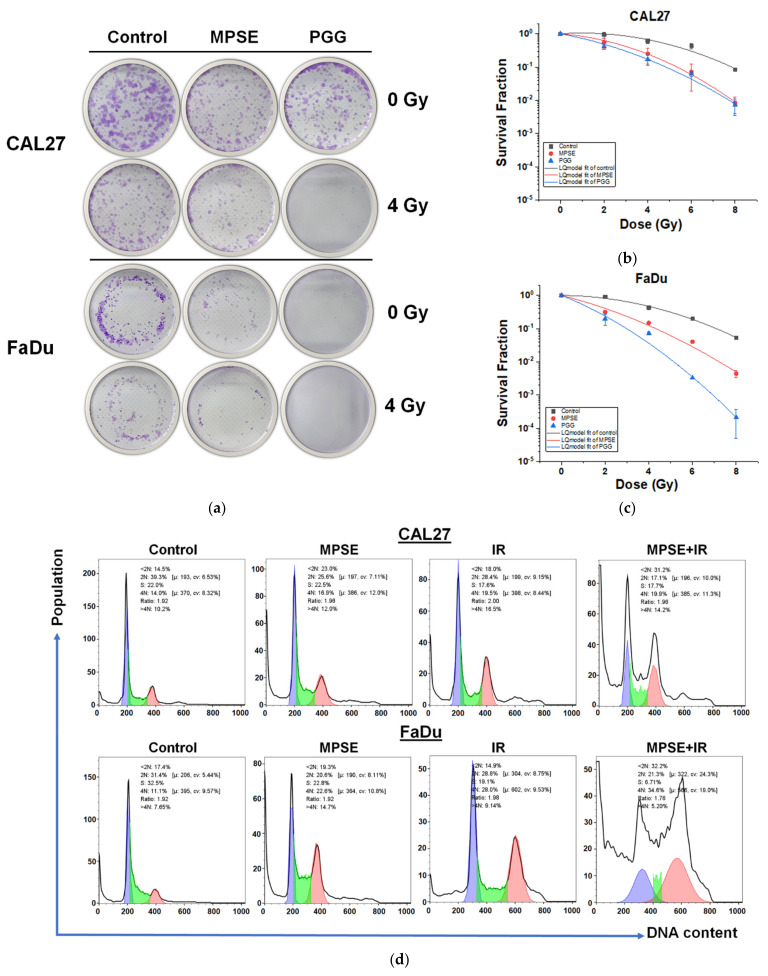

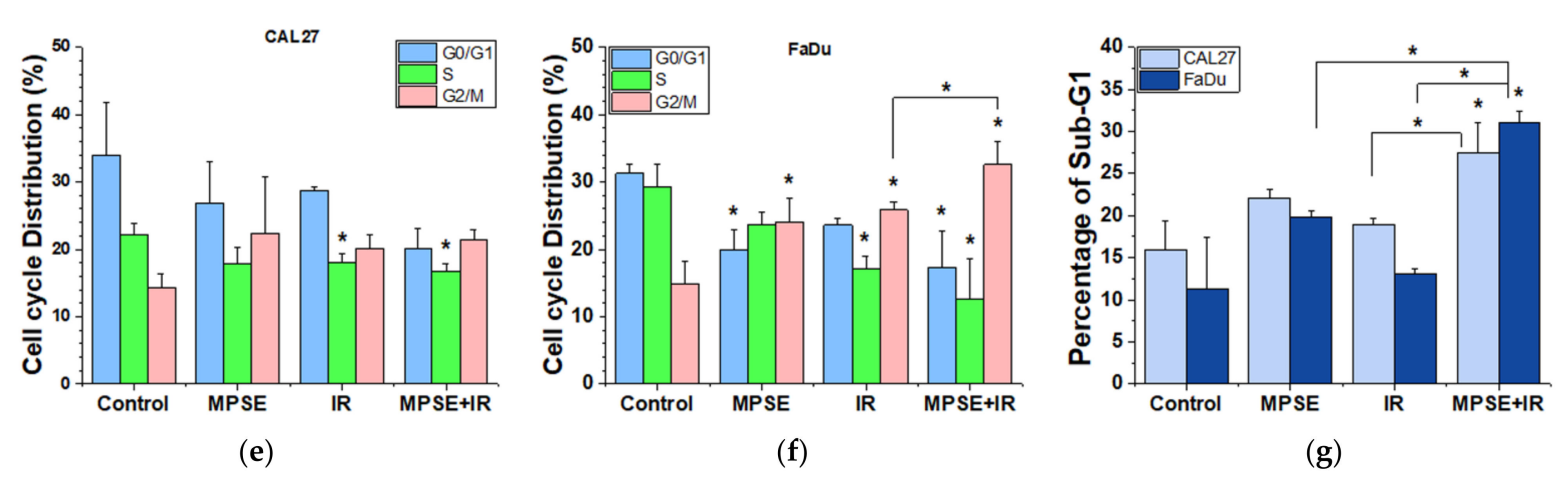
MPSE or PGG enhanced radiosensitivity and induced G2/M phase cell cycle arrest in radioresistant HPV-negative HNSCC. (**a**) Representative image of colony formation assay. (**b**,**c**) Quantitative analysis of colony formation. Radiosensitization of CAL27 and FaDu cells was analyzed in colony-forming assay after treatment with MPSE and PGG for 24 h followed by irradiation with doses of 0, 2, 4, or 8 Gy. Values are expressed as mean ± SD from three experiments. (**d**) Cell cycle distribution based on DNA content was analyzed by flow cytometry combined with propidium iodide following 24 h of indicated treatment. (**e**,**f**) The percentage of the cell population in different phases of the cell cycle was determined and plotted as a bar graph. (**g**) The percentage of a cell population in the sub-G1 phase was determined and plotted as a bar graph. All plot data show the mean ± SD (*n* = 3). Statistical significance was evaluated by one-way ANOVA, followed by post hoc Tukey’s multiple comparison test (* *p* < 0.05). MPSE, Maprang seed extract. PGG, pentagalloyl glucose. HNSCC, head and neck squamous cell carcinoma.

We further evaluated the efficacy of MPSE or PGG to increase the sensitivity of HPV-negative head and neck cancer cells, which is known for radioresistant cancer cells. CAL27 and FaDu cells were pretreated with either MPSE or PGG at a noncytotoxic dose concentration (IC_20_) of 15 µg/mL for 24 h before being irradiated (IR) with varying radiation doses from 2 to 8 Gy and then analyzed using a clonogenic assay. The typical images for colony formation from different treatments are shown in Figure 4a. We found that radiation treatment alone has very little effect on the decreased survival rate in both cancer cells. In contrast, pretreatment of CAL27 cells with MPSE or PGG for 24 h before radiation was observed to enhance their radiosensitivity, with sensitivity enhancement ratios (SER) of 1.47 and 1.62 when measured at a survival fraction level of 0.1 and following 15 µg/mL MPSE or PGG treatment, respectively (Figure 4b). Similarly, MPSE and PGG radiosensitizer FaDu cells showed this effect to a slightly higher extent, with SER of 1.65 and 2.37 when measured at a survival fraction level of 0.1 and following 15 µg/mL MPSE or PGG treatment, respectively (Figure 4c). These results suggest that MPSE or PGG pretreatment results in enhanced radiosensitivity in radioresistant HNSCC cells. To delineate the mechanism underlying the MPSE-induced radiosensitization, we further evaluated MPSE-induced cell cycle arrest in HNSCC, CAL27 and FaDu cells that were treated with irradiation alone or in combination with MPSE 15 µg/mL for 24 h and then analyzed via flow cytometry. We observed that treatment of cells with MPSE or IR alone induced G2/M phase arrest. This effect was not seen for untreated control cells. In contrast, combination treatment with MPSE and IR increased the cell population arrested in the cell cycle at the G2/M phase. Correspondence with a large proportion of the sub-G1 population was observed following combination treatment with IR and MPSE (Figure 4d). The mean percentage of G2/M cells population in untreated cells, MPSE, IR- and co-treatment with IR and MPSE-treated cells was 14.33 ± 2.02%, 22.36 ± 8.42%, 20.03 ± 2.15% and 21.47 ± 1.50% for CAL27; 14.77 ±3.51%, 23.96± 3.55%, 25.83 ± 1.25%, and 35.12 ± 0.81% for FaDu cells, respectively (Figure 4e,f). Similarly, co-treatment with IR and MPSE resulted in a further increase in the mean percentage of the sub-G1 population as compared with untreated control cells and a single treatment. The sub-G1 population increased from 16.03 ± 3.15% to 22.06 ± 1.07%, 18.86 ± 0.81%, and 27.60 ± 5.10% in CAL27 cells, and 11.33 ± 6.10 % to 19.86 ± 0.67%, 13.07 ± 0.57%, and 31.07 ± 1.25% in FaDu cells (Figure 4g).

### 2.5. MPSE or PGG Enhances Radiation-Induced DNA Damage and Potentiates Radiation-Induced Cell Death in HNSCC Cell Lines

**Figure 5:**
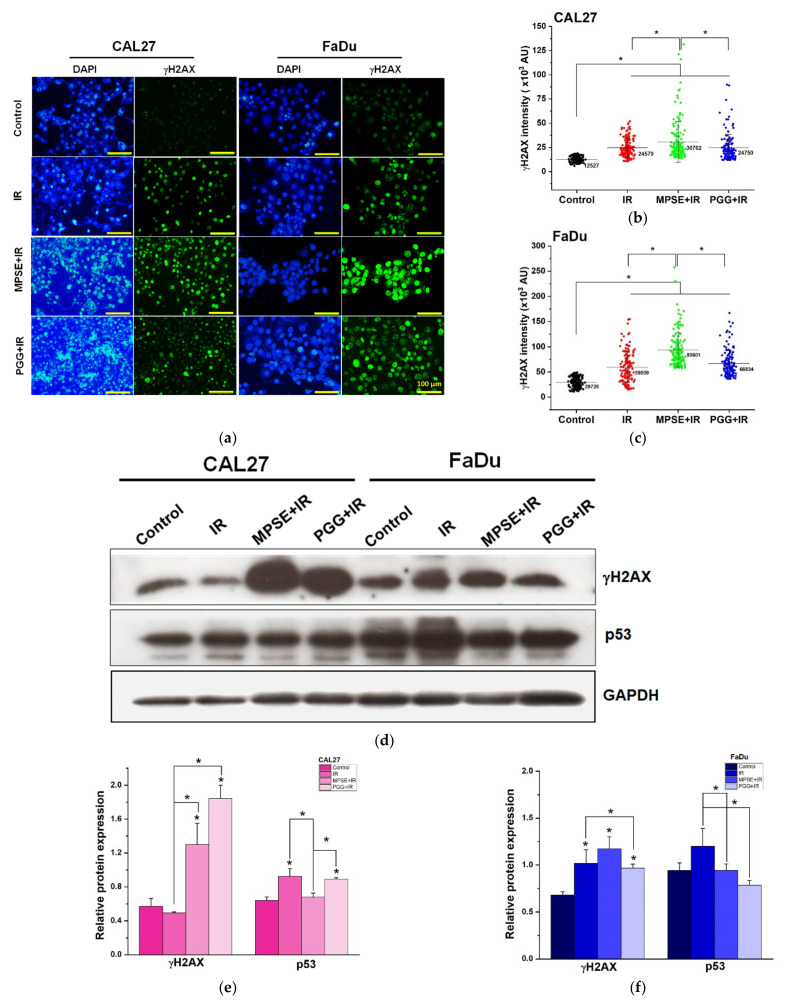

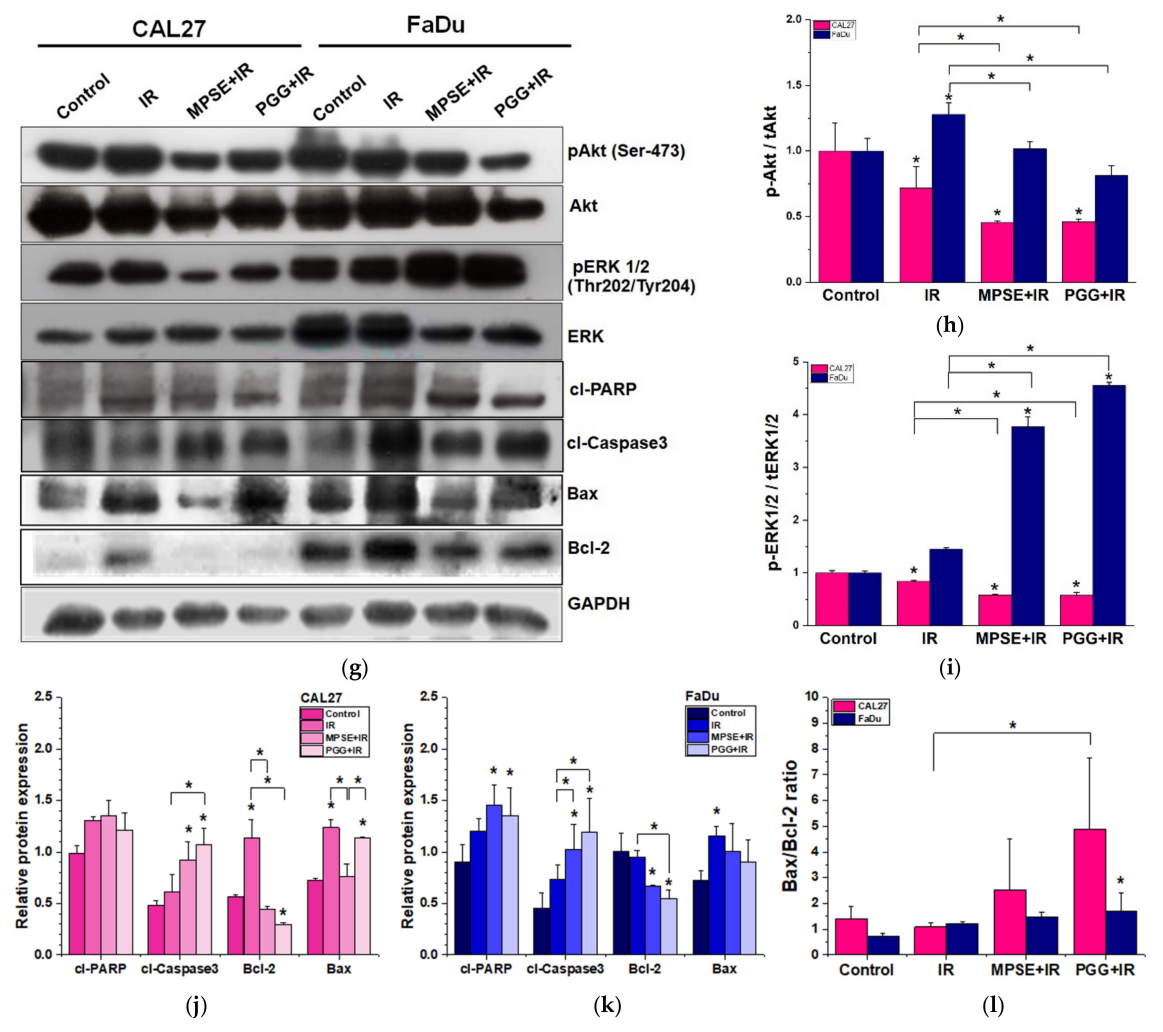

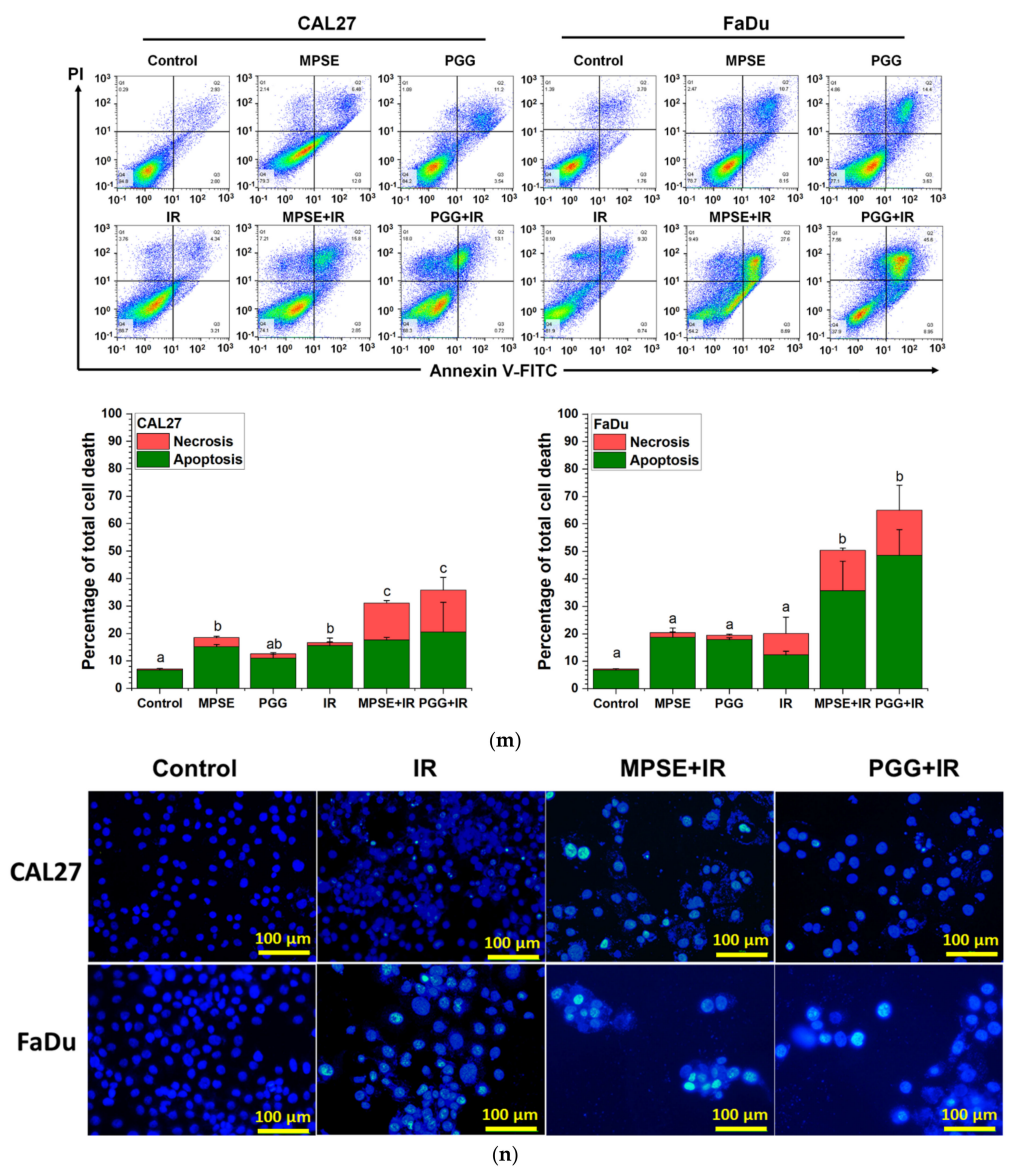
MPSE or PGG enhanced IR-induced DNA damage and cell death in HNSCC cell lines. (**a**) Representative images of γH2AX foci by immunofluorescence staining with antibodies against γH2AX for different treatments. (**b**,**c**) Quantification of γH2AX foci in (**b**) CAL27 or (**c**) FaDu cells treated with IR alone or combination of 15 µg/mL MPSE or PGG with IR. Data obtained from >100 cells (circles), numbers in the graph represent the mean of each group. (**d**) The protein expression levels of DNA damage response markers, including phosphorylated γH2AX and p53 were determined by Western blot analysis in the indicated groups. (**e**,**f**) The immunoblot signal intensities were quantified by densitometry; relative band intensity was normalized to the band intensity of GAPDH. (**g**) CAL27 and FaDu cells were treated as indicated. After 48 h, expression levels of phospho-Akt, phospho-ERK1/2, Akt, ERK, cleaved PARP, cleaved caspase3, Bax, and Bcl-2 were analyzed by Western blotting. (**h**–**k**) The immunoblot signal intensities were quantified by densitometry. The relative band intensity was normalized to the band intensity of GAPDH. (**l**) Densitometric evaluation of the expression levels of Bax/Bcl-2 proteins normalized to GAPDH and changes in Bax/Bcl-2 ratio. (**m**) Apoptosis induction was analyzed using Annexin V/PI staining after CAL27 and FaDu cells were treated as indicated. Upper: the representative flow cytometry plots are presented for each cell. Lower: quantitated total cell death after treatment with different treatment. All data are presented as mean ± SD (*n* = 3). Different letters (abc) at the top of columns indicate significant differences at *p* < 0.05 using one-way ANOVA and Tukey’s multiple comparison test. (**n**) CAL27 and FaDu cells were treated as indicated for 48 h. Cells were stained with DAPI and imaged by fluorescence microscope. All data are presented as the mean ± SD. of three independent experiments. Statistical significance was evaluated by one-way ANOVA, followed by post hoc Tukey’s multiple comparison test (* *p* < 0.05). MPSE, Maprang seed extract. PGG, pentagalloyl glucose. HNSCC, head and neck squamous cell carcinoma. IR, irradiation, DAPI, 4′,6-diamidino-2-phenylindole.

Radiotherapy efficacy mainly relies on its ability to cause lethal damage to the DNA of cancer cells. Unfortunately, one of the main factors of radioresistance is attributed to the DNA-repair potential of cancer cells. Therefore, targeting the DNA repair machinery system could enhance radiotherapy effectiveness in cancer cells. To further evaluate the effects of MPSE or PGG on IR-induced DNA damage, γH2AX foci, which represent the DNA double-stand break, in both CAL27 and FaDu cells were investigated. We performed immunofluorescence staining with antibodies against the phosphorylated histone 2A family member X (γH2AX). As shown in Figure 5a, we observed a significant increase in γH2AX staining when treated with radiation compared to untreated control cells. MPSE pretreatment resulted in further significantly enhanced γH2AX foci number in both cancer cell lines compared to radiation treated alone. In contrast, PGG pretreatment does not considerably alter the γH2AX foci number in both cancer cell lines compared to in radiation treated alone. The quantitative results are shown in Figure 5b,c. The effect of combining MPSE or PGG with IR, thereby potentiating IR-induced DNA damage, was then evaluated by Western blot analysis of the expression of DNA damage response protein. Our results revealed that the protein expression levels of γH2AX and p53 were sustained at the low levels in untreated control cells and slightly increased following IR treatment. However, the combination treatment with either MPSE or PGG and IR further increased the expression level of markers of DNA damage response protein γH2AX, while decreasing the expression of p53 in both CAL27 and FaDu cells (Figure 5d–f). These results suggest that MPSE and PGG effectively enhanced IR-induced DNA double-strand break and prolonged the damage to mediated radiosensitization in HNSCC cell lines.

It has been shown that radiation induces the activation of the pro-survival signaling, and subsequently promotes radioresistance through the DNA damage repair machinery [23]. We next examined the effects of MPSE and PGG in combination with IR on the activation of Akt and ERK1/2. The results show that IR treatment induced upregulation of Akt and ERK1/2 phosphorylation, compared with the control group. The combined treatments of either MPSE or PGG with IR could significantly downregulate basal and IR-induced Akt phosphorylation, with no significant change in the expression of total Akt in both cell lines. A less pronounced decrease of ERK1/2 phosphorylation following combination treatment was also detected for Cal27 cells. In contrast, FaDu cells showed elevated phosphorylated ERK1/2 levels in response to IR and a significant increase in ERK1/2 phosphorylation upon the treatment of MPSE and PGG combined with IR (Figure 5g–i). These results indicate that the activation of Akt and MAPK signaling most likely contribute to cellular defense in stress response to treatment and can be abolished by MPSE or PGG. To investigate the impact of the regulation of Akt and ERK1/2 phosphorylation on radiation sensitivity in the CAL27 and FaDu cells, the apoptotic proteins of cells were examined. As shown in Figure 5g,j,k, we detected an apoptosis protein marker, including the activated cleavage products of caspase-3 and PARP, pro-apoptotic protein Bax, and anti-apoptotic protein Bcl-2 by Western blot. The results revealed that in CAL27 cells, the anti-apoptotic protein Bcl-2 was markedly increased after treatment with IR alone and dramatically reduced in the combined treatment. In contrast, Bax protein expression showed a significant increase after treatment with IR and PGG combined with IR, while MPSE combined with IR treatment showed no significant changes in expression of Bax. These resulted in a significant elevation of the ratio of Bax/Bcl-2 in PGG combination with IR treatment compared to that of IR treatment alone.

Contrary to CAL27, in FaDu cells showed a higher basal level of Bcl-2. The anti-apoptotic factor Bcl-2 was markedly reduced after treatment with either MPSE or PGG combined with IR. Interestingly, the level of Bax remained the same as untreated cells in combination treatment, while IR alone treatment causes a significant increase level of Bax expression. Therefore, the ratio of Bax/Bcl-2 was elevated after combined treatment, with a significant increase in PGG combined with IR compared to untreated cells (Figure 5l). In correspondance, our results showed an increased expression level of activated cleavage of caspase-3 and PARP in the IR treatment alone group compared to untreated cells, while the combinatory treatment of either MPSE or PGG and IR was able to significantly increase its expression compared to the IR treatment alone. These results suggested that the pretreatment with either MPSE or PGG before irradiation potentiate the irradiation-induced apoptosis cell death. However, in CAL27 cells, the exact treatment dosage possessed a negligible effect compared to FaDu cells to cause increased PARP cleavage. Although, we observed the marked elevation of the ratio of Bax/Bcl-2 in combination treatment compared to that of the control and IR treatment alone.

The apoptotic activities were further quantified by flow cytometry analysis using Annexin V-FITC/PI double staining. The treatment with IR alone induced very minimal apoptosis due to intrinsic tumor radioresistance properties of both cell lines. However, pretreatment with the noncytotoxic dose concentration (IC_20_ = 15 µg/mL, at 24 h) of MPSE or PGG, at which either MPSE or PGG induced minimal apoptosis approximately 20% (Figure 5m), combination with IR tremendously increased the apoptotic activity of IR-induced cell death in both cell lines. Remarkably, in FaDu cells, the combination of low doses of MPSE or PGG and IR exhibited highly synergistic apoptotic effects. The combination of MPSE or PGG with IR exerted 2.5–3-fold more apoptosis than the additive level of apoptosis induced by a single treatment. Consistent with the observation of nuclei condensation (Figure 5n), it was revealed that the combination treatment of MPSE or PGG with radiation significantly increased the proportion of apoptotic and necrotic cell death in both cell lines.

Notably, the comparison between the two cell lines showed a strong apoptosis induction in FaDu cells compared to CAL27 cells after combination treatment. The CAL27 cells were more resistant to MPSE or PGG combined with IR-induced overall death than the FaDu cells. These results might explain the different radiosensitivity observed in a clonogenic assay. These results collectively suggest that an increase of IR-induced DNA damage that leads to enhanced IR-induced cell death may be one of the mechanisms of MPSE- and PGG-mediated radiosensitization in HNSCC cell lines.

### 2.6. Pretreatment with MPSE or PGG Attenuates Radiation-Induced Enrichment of the Stem-like Population in HNSCC Cell Lines

**Figure 6:**
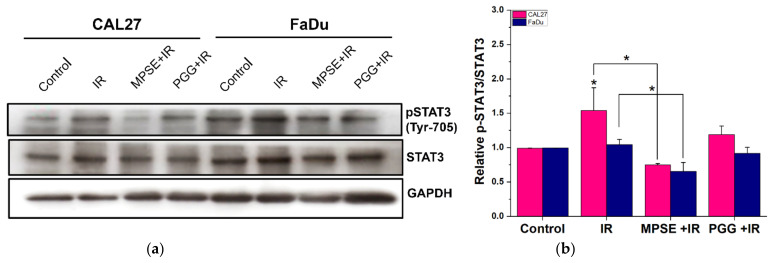

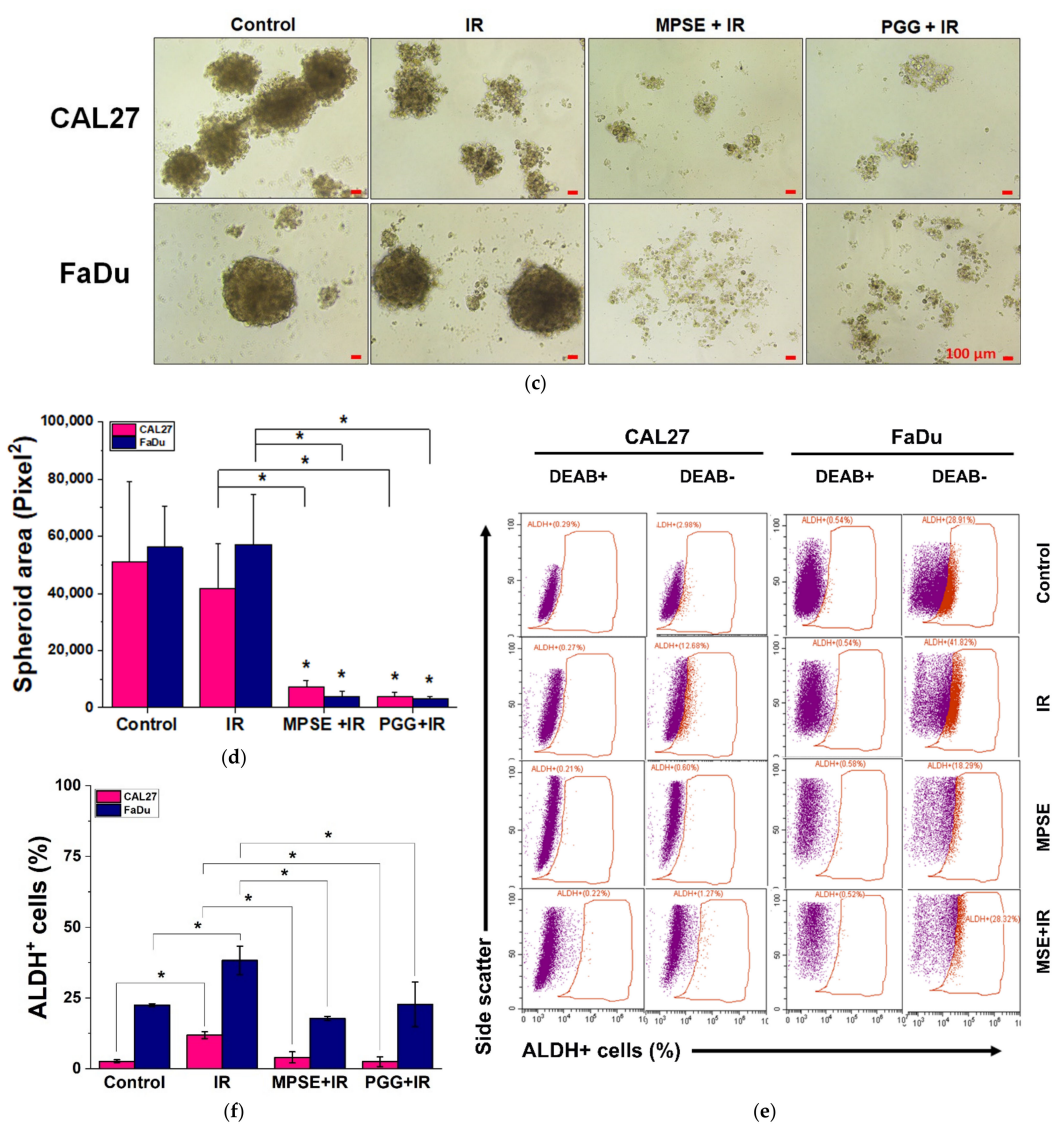

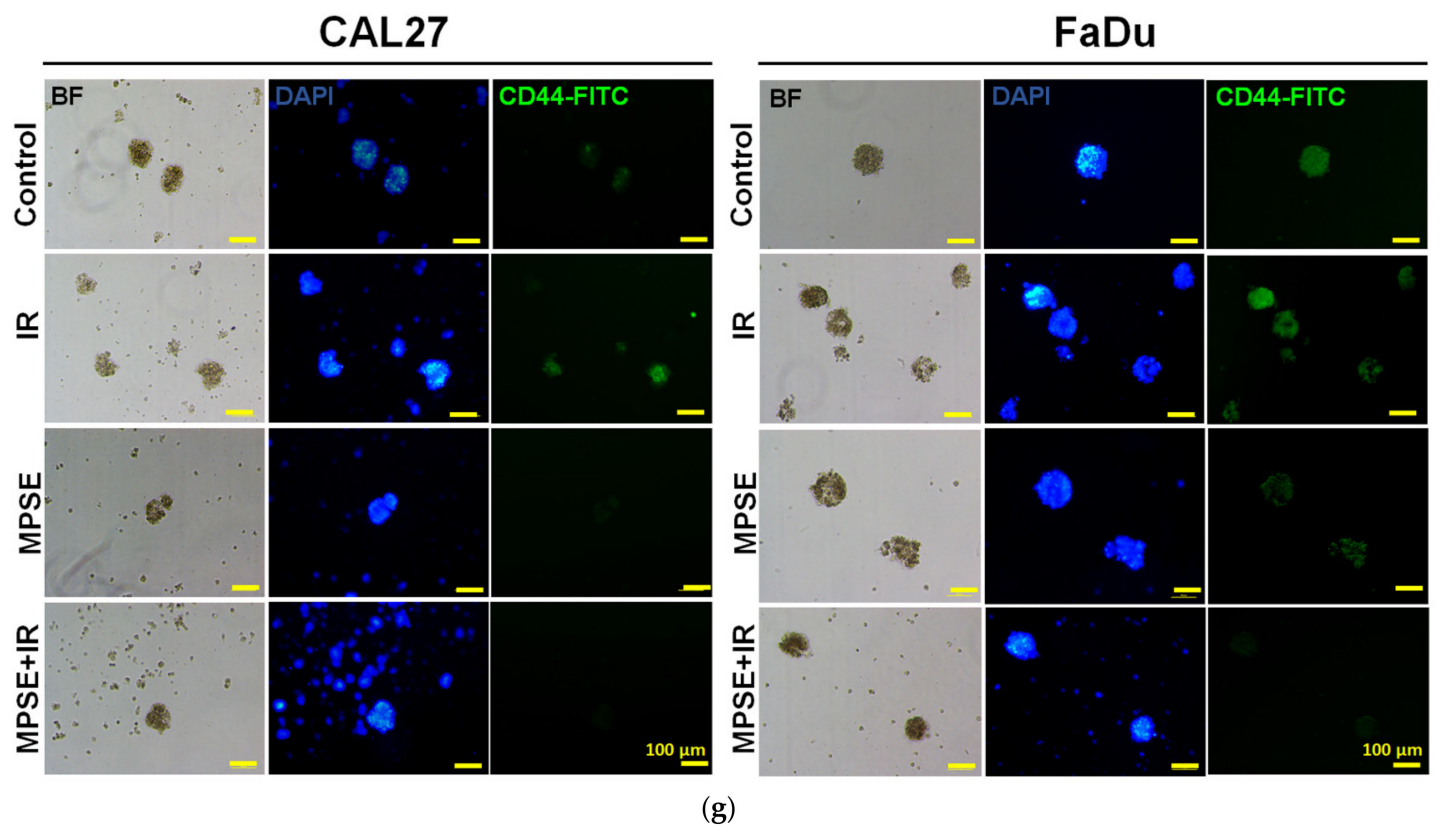
Pretreatment with MPSE or PGG before combining with irradiation suppressed the radiation-induced CSC phenotype in the HNSCC cell line. (**a**) Western blot analysis was performed to determine STAT3 phosphorylation in the indicated groups and (**b**) their band intensity was quantified. (**c**,**d**) CAL27 and FaDu cells were treated with either IR alone or the combination of 15 µg/mL MPSE or PGG with 6 Gy IR X-ray for 24 h and then were resuspended and cultured to form spheroids. Tumorsphere growth was quantified by determining the spheroid area using ImageJ software on day 10 (bar, 100 µm). (**e**,**f**) The percentages of head and neck cancer cell populations that expressed CSC markers (ALDH+). Data are presented as the mean ± SD of three independent experiments. (**g**) Immunofluorescence staining of the CSC marker (CD44) was determined in tumor spheroids. Statistical significance was evaluated by one-way ANOVA, followed by post hoc Tukey’s multiple comparison test (* *p* < 0.05). MPSE, Maprang seed extract. PGG, pentagalloyl glucose. HNSCC, head and neck squamous cell carcinoma. IR, irradiation. CSCs, cancer stem cells. ALDH, Aldehyde dehydrogenases.

Several studies suggest that radiation treatment induced an enhanced population of cancer stem cells (CSCs). CSCs have been shown to possess radio-/chemotherapy resistance and contribute to incomplete therapeutic responses of tumors, suggesting that CSCs represent a potential target for improving therapeutic efficiency. To investigate the effect of MPSE and PGG on radiation-induced CSCs and determine the role of STAT3 activation in radioresistant phenotype, we first analyzed the expression level of phosphorylated STAT3 of HNSCC cell lines, CAL27, and FaDu after treatment. We found a significantly increased phosphorylated STAT3 expression level in post-irradiation HNSCC cells as compared with untreated control cells. However, MPSE and PGG pretreatment significantly downregulated IR-induced STAT3 phosphorylation with no significant change in total STAT3 expression (Figure 6a,b). These results suggest that the activated STAT3 pathway may correlate with chemoresistance. To determine the impact of STAT3 activation on cancer stem-like cell self-renewal ability the tumor-sphere formation assay was assessed in HNSCC cell lines treated with IR alone or in combination with either MPSE or PGG. As expected, we observed that IR treatment resulted in an increased sphere formation and growth of tumorspheres. The pretreatment with MPSE or PGG significantly inhibited the formation of the tumorsphere compared to IR alone treatment (Figure 6c,d). Consistent with tumorsphere formation experiments, MPSE and PGG combination with IR significantly decreased ALDH+ cells that were enriched by irradiation treatment (Figure 6e). To further analyze the effects of STAT3 signaling in CSCs, we then assessed the expression level of the CSC marker CD44 in the tumorsphere. Corresponding with STAT3 activation, CSC markers CD44 were all diminished in the combination of either MPSE or PGG and IR treated cells (Figure 6f). Collectively, the combination of either MPSE or PGG with IR may attenuate STAT3 activation and subsequently may reduce the population of IR-induced enriched CSCs in HNSCC. These data suggested a potential application of MPSE and PGG to overcome radioresistance and improve the therapeutic outcome.

## 3. Discussion

Radiotherapy is a mainstay and an affordable treatment choice for head and neck squamous cell carcinoma (HNSCC). However, radioresistance is the main cause of treatment failure, and subsequently disease-related mortality. Therefore, the identification of molecules to strengthen therapeutic outcome is the main challenge in HNSCC treatment. In the present study, we provided insight into the mechanisms of gallotannin-rich extract compounds (which are naturally perfectly mixed constituents by 50%PGG, 36%EG, and 0.5%GA) from Maprang (*Bouea macrophylla* Griffith) seed (MPSE) and its major active compound, PGG, as a promising radiation sensitizer in HNSCC cell lines. MPSE or PGG could enhance the efficacy of radiotherapy in HNSCC via (a) inhibiting IR-induced pro-survival signaling and enhancing the effect of IR-induced DNA damage. (b) Inhibiting IR-induced enrichment of cancer stem cells population, that is responsible for radioresistance in cancer, and subsequently inhibiting anti-apoptotic pathways and increasing IR-induced cell death in HNSCC. In addition, the studies on molecular mechanisms revealed that MPSE or PGG could enhance the radiosensitivity of HNSCC via targeting cancer stem-like cells through attenuated STAT3 activation.

We have recently demonstrated that MPSE possessed promising anti-cancer activity and radiosensitization in breast cancer cells by inhibiting IR-induced pro-survival signaling [19]. This study extends this observation to HNSCC, where poor patient prognosis and treatment failure were found due to intrinsic tumor radioresistance in HNSCC. In this study, we tested the activity of either MPSE or PGG in two radioresistant HPV-negative HNSCC (FaDu and CAL27) cells [24]. Here, we demonstrated that MPSE or PGG suppresses the proliferation of HNSCC cells. In a comparison between two HNSCC cell lines, we also observed that FaDu cells are more responsive to MPSE than CAL27 cells (Figure 2). Since HPV-negative HNSCC cells are incredibly resistant to IR [25], we tested whether MPSE or PGG radiosensitizes CAL27 or FaDu cells. In line with the above finding, strongly decreased clonogenicity and increased cell death were seen after combined treatment with either MPSE or PGG and IR found in FaDu compared with CAL27 cell lines (Figure 4 and Figure 5). The combined treatment with either MPSE or PGG and IR exerts a negligible effect on enhanced apoptosis cell death in CAL27 cells (Figure 5m). However, the significant complete loss of clonogenic potential was observed in combination treated CAL27 cells (Figure 4b) through a possible induction of non-apoptotic programmed cell death. Thus, the exact nature of the programmed cell death, other than apoptosis, after the combination treatment of MPSE and IR in CAL27 cell line remains unclear and requires further investigation.

It has been proposed that the presence of a small population of CSCs in tumors results in resistance and recurrence of cancer after therapy [3]. Therefore, potential compounds that can eliminate CSCs are imperative to overcome therapeutic resistance and improve the clinical outcomes of patients. The activation of STAT3, which is known to increase survival and proliferate signals, has been shown to play a critical role in regulating stemness in many cells, especially in HNSCC [10]. To maintain CSC characteristics, STAT3 regulates the expression of several downstream target proteins associated with the stemness properties. Many CSC proteins and markers downstream of STAT3 have been identified, such as CD44, CD133, ALDH1, and the transcription factor regulates CSC (Oct4 and Sox2). Moreover, CSC self-renewal properties were shown to depend on STAT3 activation [26]. Highly activated STAT3 is positively correlated with high-grade HNSCC tissue and promotes CSCs’ self-renewal, and the radioresistance ability of HNSCC. Blocking STAT3 activation by a specific inhibitor effectively blocked the tumor spheroid formation and decreased the number of CD44+ ALDH+ cells, and further induced apoptosis in HNSCC [13], this was consistent with our study. We found that the inhibition of STAT3 by MPSE or PGG could suppress CSC in FaDu and CAL27 cells. For HNSCC cells, either adherent or spheroid-derived CSC-rich population treatment with MPSE or PGG was shown to downregulate the expression of phosphorylated STAT3. Subsequently, it downregulated CSC-related proteins, Oct4, SOX2, and CD44. Moreover, MPSE or PGG treatment dramatically suppressed their spheroid formation ability (Figure 3). This is linked to the effect of MPSE or PGG in suppressing activities on key stem cell-related and oncogenic pathway STAT3 signaling.

Following radiation treatment, the occurrence of DNA damage, especially to double-strand breaks (DSBs), has resulted in cell cycle arrest, apoptosis, and tumor regression. However, to survive, tumor cells always activate the pro-survival pathway and DNA repair mechanisms such as PI3K/Akt or RAS/Raf/Erk1/2 [27]. Thus, tumor cells with highly efficient DNA repair are radioresistant [28], whereas impaired repair DSBs are particularly detrimental to the cells. Recently, we have shown that MPSE increases IR-induced H2AX foci. Moreover, MPSE radiosensitized breast cancer cell lines via the inhibition of the pro-survival PI3K/Akt pathway. Similarly, in this study, we noticed that radiosensitization induced by MPSE or PGG was associated with impaired DNA repair, as evidenced by the marked decrease in the IR-induced phosphorylation of Akt and phosphorylation of ERK1/2, and increase in DNA damage response protein, γH2AX in HNSCC cells (Figure 5). This implies that the DNA repair pathway, in the path, plays a critical role in the regulation of MPSE- or PGG-mediated radiosensitization in HNSCC cell lines.

Recent evidence indicates that IR may induce the generation of a new CSCs population during a radiotherapy course [29]. As previously mentioned, a critical event that determines the cancer radioresistance is CSCs [3]. Suppression of IR-induced CSCs could enhance the efficacy of radiation therapy in human cancer cells. Our studies demonstrate that MPSE or PGG effectively suppress the expression of CSCs in HNSCC cells. We further analyzed the effect of IR on CSCs. As expected, we found that IR increased the number of CSCs in both FaDu and CAL27 cells. It worth nothing that pretreatment with MPSE or PGG significantly reduced the proportion of CSCs in comparison to either IR alone or untreated control (Figure 6). These results suggest that pretreatment of MPSE or PGG before IR might eradicate both CSC and non-CSC populations, and this could prevent tumor recurrence.

## 4. Materials and Methods

### 4.1. Chemicals and Reagents

Eagle’s Minimum Essential Medium (EMEM) and Dulbecco’s Modified Eagle’s Medium/Nutrient Mixture F-12 (DMEM/F-12) were purchased from Caisson Lab (Smithfield, UT, USA). Trypsin-EDTA, fetal bovine serum, penicillin, and streptomycin were purchased from Gibco Thermo Fisher Scientific (Waltham, MA, USA). 3-(4,5-dimethylthiazol-2-yl)-2,5-diphenyltetrazolium bromide (MTT), bovine serum albumin (BSA), human insulin, epidermal growth factor, basic fibroblast growth factor, hydrocortisone, penta-*O*-galloyl-β-*D*-glucose hydrate (PGG), ethyl gallate (EG), and gallic acid (GA) were purchased from Sigma-Aldrich (St. Louis, MO, USA). The annexin V-FITC/PI apoptosis detection kit and AldeRed ALDH detection assay were also purchased from Sigma-Aldrich (St. Louis, MO, USA). PMSF and cocktail protease inhibitors were purchased from Hi Media Laboratories (Marg, Mumbai, India). Additionally, 4′,6-diamidino-2-phenylindole, dihydrochloride (DAPI) was obtained from Thermo Fisher Scientific (Waltham, MA, USA). Primary antibodies against total Akt, phosphorylated Akt (Ser473), total ERK1/2, phosphorylated ERK1/2(Thr202/Tyr204, Thr185/Tyr187)) total STAT3, phosphorylated STAT3 (Tyr705), cleaved caspase 3, Bcl2, cleaved PARP, CD44 and GAPDH, as well as horseradish-peroxidase-labeled secondary antibodies, were purchased from Merck (Merck KGaA, Darmstadt, Germany). Anti Oct4 monoclonal antibody was obtained from Invitrogen (Waltham, MA, USA). Anti Sox2 antibody was from Thermo Fisher Scientific (Waltham, MA, USA). Primary rabbit polyclonal antibodies against γH2AX and Alexa Fluor 488 conjugated rabbit anti-human IgG antibodies were from Thermo Fisher Scientific (Waltham, MA, USA).

### 4.2. MPSE Preparation, Isolation of PGG, and High-Performance Liquid Chromatography (HPLC) Quantification of Major Active Ingredients

*Bouea macrophylla* Griffith (Maprang) seeds were collected with permission from private landowners from the Maprang Plantation located in Nakhon Nayok Province. The plant was authenticated by Dr. Tanawat Chaowasku, and the voucher specimens (CMUB39942) of these plants have been deposited at the CMUB herbarium at Chiang Mai University, Thailand. Dried seed kernel was prepared as described by previous work [15,16] with a modification. In brief, 50 g of the minced dried seed was immersed in 500 mL of 75% ethanol to macerate them for 7 days with daily shaking. Then, the extracts were filtered, and ethanol was evaporated in a rotatory evaporator system (Buchi Rotavapor R-100, BÜCHI Labortechnik AG, Flawil, Switzerland). After lyophilization, a total of 20 g of extract powder (MPSE) was obtained (40% yield). MPSE was collected and stored at room temperature in a desiccator for further study. To prepare the stock solution, MPSE were solubilized with deionized water (Pure Lap Option-Q, ELGA LabWater, UK) at a final concentration of 1 mg/mL. The solution was then filtered with a 0.22 µm syringe filter and the extract active gradients, penta-*O*-galloyl-β-*D*-glucose hydrate (PGG), ethyl gallate (EG), and gallic acid (GA) were quantified by using Shimadzu LC-20AD Prominence Liquid Chromatograph system equipped with a SPD-M20A Prominence Diode Array Detector (Shimadzu, Nakagyo-ku, Kyoto, Japan) according to a previously well established and validated method [15]. To isolate PGG, 1 g/L MPSE in deionized water was prepared and kept at −20 °C in a freezer for 3 h and then cooled to 4 °C by air-exposure at room temperature for 3 h. After that, the cold solution was collected in a 50 mL centrifuge tube and centrifuged at 2500× *g* for 20 min (NUVE NF400; Nuve Sanayi Malzemeleri Imalat TAS, Akyurt, Ankara, Turkey). The pellets were washed with 50 mL cold water and centrifuged at 2500× *g* for 20 min for 5 cycle times. Next, the PGG pellets were collected and lyophilized to powder (weight = 0.1322 g; yield = 13.22%) and kept in a desiccator at room temperature for further study. PGG purity was 97% determined by HPLC. 

### 4.3. Cell Lines and Cell Culture Conditions

Human head and neck squamous cell carcinoma CAL27 and FaDu cell lines were obtained from American Type Culture Collection (Manassas, VA, USA). CAL27 and FaDu were maintained in Eagle’s Minimum Essential medium (EMEM) supplemented with 10% fetal bovine serum (FBS), 2 mM l-glutamine, and 100 U/mL penicillin and streptomycin. Cell cultures were maintained at 37 °C and with a 95% air humidified incubator with 5% CO_2_.

### 4.4. Cell Proliferation Assay

Cells were seeded onto 96-well plates at a density of 5 × 10^3^ cells/well and allowed to grow overnight in an incubator. Cells were then treated with various concentrations of MPSE or PGG (0–100 µg/mL) for an appropriate time (24, 48, and 72 h). Cell viability was assessed by 3-(4,5-dimethylthiazol-2-yl)-2,5-diphenyltetrazolium bromide (MTT) assay, in brief, cells were incubated with MTT solution (0.1 mg/mL) for 4 h in a 37 °C incubator and then the formazan product was solubilized using 100 µL of DMSO. The intensity of the formazan solution was determined by measurement of the absorbance at 560 nm using a microplate reader (BioTekTM Eon^TM^ microplate reader, Winooski, VT, USA). Relative cell viability was calculated by dividing the absorbance of the treated cells by that of the control cells. The half-maximal inhibition concentration (IC_50_) was determined from three independent experiments using OriginPro version 2018 software (Origin Lab, MA, USA).

### 4.5. Irradiation Treatment

Cells were grown in a T75 cm^2^ cell culture flask and treated with or without either MPSE or PGG for 24 h and then subjected to a single dose of irradiation (IR). IR was performed using a linear 6 MV X-ray accelerator at a dose rate of 200 MU/minute (Primus, Siemens Healthineers, Malvern, PA, USA) at room temperature.

### 4.6. Clonogenic Assay

Cancer cells were seeded in 6-well plates and allowed to grow overnight, then treated with or without MPSE or PGG (pretreatment) for 24 h and irradiated with various doses of radiation (2–8 Gy). Cells were maintained for 14 days to allow colonies to form. After that, colonies were fixed in fixation solution (3:1 of methanol/acetic acid) for 30 min at room temperature and stained with 1% crystal violet. Cell colonies with more than 50 cells were counted using an inverted microscope (ECLIPSE Ts2, Nikon, Tokyo, Japan). Plating efficiency was calculated by dividing the average number of colonies per well by the number of cells plated. Survival fractions were calculated by normalization to the plating efficiency of appropriate control groups. These data were used for survival curve plotting and fitting as a function of X-ray dosage using a linear-quadratic model via OriginPro version 2018 software (Origin Lab, MA, USA).

### 4.7. Cell Cycle Analysis

Changes in cell cycle distribution induced by either IR or MPSE (15 µg/mL) combined with IR were analyzed using propidium iodide (PI) staining. In brief, cells were seeded into 6-well plates and treated as indicated then cells were incubated for 24 h. After completion of the incubation phase, both the suspension cells and the adherent cells were harvested, washed with PBS, and centrifuged at 7000 rpm for 1 min. The supernatant was discarded, and the pellets were collected and then fixed with 70% ethanol overnight at 4 °C. Subsequently, the fixed cells were washed with ice-cold PBS before being stained with the binding buffer containing 0.1% Triton X-100, 0.2 mg/mL of RNase, and 10 µg/mL of PI. Stained cells were then incubated at 37 °C for 30 min in the dark. Finally, the stained cells were analyzed by flow cytometry (Epics XL-MCL, Beckman Coulter, Brea, CA, USA). Flow cytometric data were then analyzed by FlowJo™ software version 10 (Becton, Dickinson & Company, NJ, USA; https://www.flowjo.com/solutions/flowjo/ accessed on 16 January 2021).

### 4.8. Spheroid Formation Assay

Cells were treated as indicated and then were dissociated into single cells with 0.5 % trypsin-EDTA. Cells 5 × 10^3^ cells/well were seeded onto a 24-well ultralow attachment plate in serum-free DMEM/F-12 (1:1 ratio) media supplemented with 1% BSA, 5 µg/mL insulin, 25 ng/mL basic fibroblast growth factor, 25 ng/mL epidermal growth factor, and 0.5 µg/mL hydrocortisone. Cells were allowed to form tumorspheres for 10 days. Spheroid formation was observed and imaged using a phase-contrast microscope (ECLIPSE Ts2, Nikon, Tokyo, Japan). The area and number of tumorspheres larger than 50 µm in diameter were quantified using ImageJ 1.52v software (https://imagej.nih.gov/ij/download/ accessed on 10 March 2021).

### 4.9. Detection of Cancer Stem Cell Marker via AldeRed ALDH Assay

Cells were seeded into a 6-well plate for 24 h to allow attachment before being treated with either MPSE or PGG, IR alone, or MPSE or PGG combined with 6 Gy IR X-ray. Cancer stem cell markers were determined by AldeRed ALDH detection assay following the manufacturer’s protocol with only a few modifications. In brief, 5 × 10^5^ cells were resuspended in 1 mL AldeRed assay buffer. After the addition of 5 µL AldeRed reagent and mild mixing, 500 µL of the cell suspension was immediately transferred to another tube supplemented with 5 µL DEAB and pipetted to mix evenly. Then, both test and control tubes were incubated in a 37 °C incubator for 30 min to allow the reaction to occur. After 30 min, cells were washed twice with 2 mL AldeRed assay buffer and eventually resuspended in 500 µL AldeRed assay buffer before being analyzed by flow cytometry (CytoFLEX, Beckman Coulter, Brea, CA, USA).

### 4.10. Apoptosis Assay

Cells were seeded into a 6-well plate for 24 h to allow attachment before being treated with varying concentrations of either MPSE or PGG. Cells were harvested by trypsinization at 48 h after treatment. The harvested cells were stained using annexin V-FITC/PI apoptosis detection kit following the manufacturer’s instructions. Briefly, the harvested cells were washed by 1× binding buffer, then centrifuged at 7000 rpm for one minute and the supernatant was removed. The pellets were stained by adding 1× binding buffer, annexin V-FITC, and propidium iodide (PI) for 20 min at room temperature in total darkness. The stained cells were immediately analyzed by flow cytometry (CytoFLEX, Beckman Coulter, Brea, CA, USA). Flow cytometric data were then analyzed by FlowJo™ software version 10 (Becton, Dickinson & Company, NJ, USA; https://www.flowjo.com/solutions/flowjo/ accessed on 16 January 2021).

### 4.11. Morphological Detection of Apoptosis by DAPI Staining

Morphological characteristics of apoptotic cells were determined using 4′,6-diamidino-2-phenylindole, dihydrochloride (DAPI) staining. In brief, 2 × 10^5^ cells were seeded in 6-well plates and cultured for 24 h to allow cell attachment to occur. Cells were treated with either IR alone, or MPSE or PGG combined with 6 Gy IR X-ray for 48 h. Following treatment, the cells were washed with PBS, and then fixed with 4% formaldehyde in PBS for 15 min at room temperature and then washed twice with PBS. Cells were permeabilized with permeabilization buffer containing 1% BSA, 0.2% Triton-X100 in PBS for 1 h before being incubated with 300 nM DAPI stain solution for 5 min. Cells were protected from light throughout the procedure. The cells were washed twice with PBS and photographed with a fluorescence microscope (ECLIPSE Ts2, Nikon, Tokyo, Japan).

### 4.12. γH2AX Immunostaining Assay

Cells were plated in chamber slides and pretreated with MPSE or PGG for 24 h before irradiation with 6 Gy X-ray. At 48 h post-irradiation, the cells were fixed with 4% paraformaldehyde for 15 min at room temperature. Then, cells were permeabilized with 0.3% TritonX-100 and blocked with 5% BSA in PBS solution at room temperature for 30 min. The cells were then incubated overnight at 4 °C with primary rabbit polyclonal antibodies against γH2AX (1:500). After primary antibody incubation, the cells were washed with PBS with Tween 20 (PBST) and incubated with 1:200 diluted Alexa Fluor 488 conjugated rabbit anti-human IgG antibodies (Invitrogen, Thermo Fisher Scientific, Waltham, MA, USA) for 1 h at room temperature. Nuclei were counterstained with 4′,6-diamidino-2-phenylindole, dihydrochloride (DAPI, 1 µg/mL). Pictures were captured with an inverted fluorescence microscope (ECLIPSE Ts2, Nikon, Tokyo, Japan). γH2AX intensity was quantified using an in-house software code developed in the MATLAB (MathWorks, MA, USA) programming kindly generated by Dr. Kittichai Wantanajittikul.

### 4.13. Immunostaining of Cancer Stem Cells Markers (CD44)

After treatment, cells were allowed to form tumor spheroids as described above, and then tumorspheres were fixed with 4% paraformaldehyde for 30 min and permeabilized with 0.1% Triton X-100 for 20 min. The tumorspheres were then blocked by incubating with 3% bovine serum albumin for 30 min and further washed twice with phosphate-buffered saline (PBS) and incubated with antiCD44-FITC-conjugated dyes for 1 h, at 4°C in the dark. The cells were washed with phosphate-buffered saline and co-stained with 4′,6-diamidino-2-phenylindole (DAPI) dye and were then visualized and imaged by fluorescence microscopy (ECLIPSE Ts2, Nikon, Tokyo, Japan).

### 4.14. Western Blot Analysis

Following treatment, cells or spheroids were lysed for protein extraction by CelLytic M lysis buffer (Sigma-Aldrich, St. Louis, MO, USA) in the presence of 1% protease inhibitor cocktail for 30 min on ice. The protein contents were evaluated by Bradford assay (Sigma-Aldrich). Denatured protein samples with equal amounts of protein (15 µg) were resolved by either 10% SDS-PAGE or NUPAGETM 4–12% Bis-Tris Gels (Thermo Fisher Scientific, Waltham, MA, USA) and then the resolved proteins were transferred into PVDF membrane (Merck KGaA, Darmstadt, Germany). Further, the whole transferred membranes were cut at the appropriate molecular weight range of protein interested and blocked for 1 h in 5% non-fat dry milk in Tris-buffered saline with Tween and incubated overnight with specific primary antibodies against total AKT, phosphorylated AKT, total ERK1/2, phosphorylated ERK1/2, total STAT3, phosphorylated STAT3, SOX2, Oct4, CD44, γH2AX, as well as GAPDH. Then, membranes were washed, followed by incubation with appropriate horseradish-peroxidase-labeled secondary antibodies (at dilution 1:10,000) for 1 h at room temperature. Signals were developed using the enhanced chemiluminescence assay (Millipore Corporation) and exposed to film. The images were scanned, and the intensity of each band was captured using an Image master 2D platinum version 5.0 (GE Healthcare Amersham Bioscience, Chicago, IL, USA). The protein content was normalized to GAPDH.

### 4.15. Statistical Analysis

Data are expressed as mean ± standard deviations (SDs) of at least three independent experiments. The differences in the mean values were determined by one-way ANOVA followed by Tukey’s HSD test or Student’s *t*-test using IBM^®^ SPSS^®^ Statistics Subscription software (IBM Corp. in Armonk, NY, USA). A value of *p* < 0.05 was considered statistically significant.

## 5. Conclusions

This study provides evidence that MPSE and its isolated major bioactive compound, PGG from *Bouea macrophylla* Griffith seeds extract, have the potential to suppress cancer stem-like cells and confer enhanced radiosensitivity in HPV-negative HNSCC cells. The mechanism by which MPSE or PGG stopped the stemness trait involves the inhibition of the Akt/STAT3 pathways and subsequently decreased expression of CSC markers and CSC self-renewal. As CSCs are closely linked to the radioresistance of cancers, MPSE or PGG attenuates the IR-induced cancer stemness phenotype. The possible molecular mechanisms might be suppressing phosphorylated Akt and phosphorylated ERK1/2 due to the suppression of IR-induced pro-survival signaling and increased DNA damage leading to radiosensitization in the radioresistant HPV-negative HNSCC cell lines. Our study may hold great promise for increasing the efficacy of radiotherapy in HNSCC patients using a safe and low-cost natural product.

## Data Availability

The data presented in this study are available on request from the corresponding author.
